# Comparative analysis of retinal vascular structural parameters in populations with different glucose metabolism status based on color fundus photography and artificial intelligence

**DOI:** 10.3389/fcell.2025.1550176

**Published:** 2025-02-03

**Authors:** Naimei Chen, Zhentao Zhu, Di Gong, Xinrong Xu, Xinya Hu, Weihua Yang

**Affiliations:** ^1^ Department of Ophthalmology, Affiliated Hospital of Nanjing University of Chinese Medicine, Nanjing, China; ^2^ Department of Ophthalmology, Huaian Hospital of Huaian City, Huaian, China; ^3^ Shenzhen Eye Hospital, Shenzhen Eye Medical Center, Southern Medical University, Shenzhen, China

**Keywords:** color fundus photography, retinal vascular parameters, biomarkers, glucose metabolism status, artificial intelligence

## Abstract

**Objective:**

Measure and analyze retinal vascular parameters in individuals with varying glucose metabolism, explore preclinical retinal microstructure changes related to diabetic retinopathy (DR), and assess glucose metabolism’s impact on retinal structure.

**Methods:**

The study employed a cross-sectional design encompassing a 4-year period from 2020 to 2024. Fundus photographs from 320 individuals (2020–2024) were categorized into non-diabetes, pre-diabetes, type 2 diabetes mellitus (T2DM) without DR, and T2DM with mild-to-moderate non-proliferative DR (NPDR) groups. An artificial intelligence (AI)-based automatic measurement system was used to quantify retinal blood vessels in the fundus color photographic images, enabling inter-group parameter comparison and analysis of significant differences.

**Results:**

Between January 2020 and June 2024, fundus color photographs were collected from 320 individuals and categorized into four groups: non-diabetes (n = 54), pre-diabetes (n = 71), T2DM without overt DR (n = 144), and T2DM with mild-to-moderate NPDR (n = 51). In pairwise comparisons among individuals with pre-diabetes, T2DM without DR, and T2DM with mild-to-moderate NPDR. Fasting blood glucose (FBG), glycated hemoglobin (HbA1c), systolic blood pressure (SBP), and diastolic blood pressure (DBP) were significantly different (*P* < 0.05). Within the T2DM population, FBG, HbA1c, age, SBP, and DBP were significant predictors for mild-to-moderate NPDR (*P* < 0.05). Average venous branching number (branch_avg_v) was significantly different among pre-diabetes, T2DM without DR, and T2DM with mild-to-moderate NPDR groups. In patients with T2DM with mild-to-moderate NPDR, Average length of arteries (length_avg_a) and average length of veins (length_avg_v) increased, whereas branch_avg_v, average venous branching angle (angle_avg_v), average venous branching asymmetry (asymmetry_avg_v),overall length density (vessel_length_density), and vessel area density (vessel_density) decreased significantly (*P* < 0.05). Logistic regression analysis identified length_avg_a, branch_avg_v, angle_avg_v, asymmetry_avg_v, vessel_length_density, and vessel_density as independent predictors of mild-to-moderate NPDR in patients with T2DM. Receiver Operating Characteristic (ROC) curve analysis demonstrated that length_avg_a, length_avg_v, branch_avg_v, angle_avg_v, asymmetry_avg_v, vessel_length_density, and vessel_density had diagnostic value for mild-to-moderate NPDR (*P* < 0.05).

**Conclusion:**

In individuals diagnosed with T2DM, specific retinal vascular parameters, such as branch_avg_v and vessel_density, demonstrate a significant correlation with mild-to-moderate NPDR. These parameters hold promise as preclinical biomarkers for detecting vascular abnormalities associated with DR.

## 1 Introduction

Diabetes mellitus (DM) is one of the diseases with the most rapidly increasing incidence globally, and it is projected that 783.2 million adults will be diagnosed by 2045 (Sun C. et al., 2022). Diabetic retinopathy (DR), a common ocular complication of diabetes, has emerged as a primary causes of vision loss among the global workforce. Statistics indicate that approximately one-third of patients with diabeties will develop DR (Teo et al., 2021)), and of particular concern, approximately one in ten of these patients with DR will experience severe visual impairment (Ipp et al., 2021; Sun et al., 2021). Often, patients with diabetes experience no symptoms during the early stages of progressive pathological changes in the retina. However, by the time obvious DR changes occur, the visual function of patients will have typically already been severely compromised. Despite numerous advancements in the treatment of DR, retinal damage is usually irreversible (Flaxel et al., 2020). However, DR is a preventable disease (Teo et al., 2021). Through early standardized diagnosis and treatment, we can, to a certain extent, prevent the occurrence of DR and slow its progression (American Diabetes Association Professional Practice Committee, 2022). However, DR is a preventable disease (Teo et al., 2021). Through early standardized diagnosis and treatment, we can, to a certain extent, prevent the occurrence of DR and slow its progression (Lin et al., 2021).

Numerous studies have indicated that structural and functional alterations in the microvasculature and neural tissues of the ocular fundus occur in patients with diabetes prior to the clinical detection of DR (Chai et al., 2022). However, research on the early retinal vasculature and nerves in individuals with abnormal blood glucose levels is relatively scarce, and the vascular parameters in existing studies are limited, hindering comprehensive and integrated comparisons. Fundus color photography, characterized by its non-invasiveness, rapid imaging, high resolution, and ease of storage and transmission of records, is a convenient tool for long-term patient follow-up. Most primary hospitals are equipped with this instrument. By comparing fundus color photographs taken at different time points, precise assessments of disease progression can be made, providing a solid basis for formulating and adjusting treatment plans. Although optical coherence tomography angiography (OCTA) technology has demonstrated potential in early retinal vasculature studies of patients with DR (Waheed et al., 2023), its high cost limits widespread adoption. In contrast, the application of AI for segmentation and quantitative analysis of retinal vessels in fundus color photographs not only enables more precise quantitative assessments of the retinal vasculature system (Yang et al., 2023), enhancing the accuracy of related research, but also boasts high popularity and operational convenience, thereby demonstrating greater practical value in clinical practice (Xu and Yang, 2023; Chen et al., 2024).

This study aims to quantify retinal vascular parameters in fundus color photographs of healthy individuals and those with abnormal glucose metabolism, including in patients with pre-diabetes, T2DM without DR changes, and T2DM with mild-to-moderate NPDR. By measuring and comparing the retinal vascular morphometric parameters in patients with different glucose metabolism status, this study sought to identify biomarkers that may serve as early indicators of microvascular changes in populations with abnormal glucose metabolism. This study will provide novel insights for the diagnosis and follow-up assessment of pre-clinical DR, offering a rationale for more reasonable and efficient diagnostic and follow-up protocols. By doing so, it has the potential to reduce unnecessary medical expenditures, enable more precise disease monitoring, decrease the risk of blindness for patients, alleviate economic burdens on individuals, families, and society, and ultimately enhance quality of life.

## 2 Materials and methods

### 2.1 General information

Participants were recruited from the Health Management Center of Peking University Shenzhen Hospital, as well as the Ophthalmology and Endocrinology Departments of Huaian Hospital of Huaian City, between January 2020 and June 2024. The inclusion criteria included individuals who were willing to undergo fundus color photography and fell into one of three groups: those without diabetes, those with prediabetes, and those with type 2 DM (T2DM). All eligible participants underwent a series of examinations, including vision testing, slit-lamp examination, direct ophthalmoscopy, and fundus color photography. Subsequently, based on the results of the fundus color photography findings, the study population was categorized into four groups: patients without diabetes (n = 54), with prediabetes (n = 71), with T2DM without significant DR changes (n = 144), and with T2DM accompanied by mild to moderate NPDR changes (n = 51). A total of 320 participants were enrolled in this study. The study protocol was approved by the Medical Ethics Committees of both Peking University Shenzhen Hospital and Huaian Hospital of Huaian City.

### 2.2 Diagnostic criteria

#### 2.2.1 Diagnostic criteria for prediabetes

In line with China’s national conditions and with reference to the blood glucose diagnostic criteria from the 2020 edition of the “Guidelines for Prevention and Treatment of Type 2 Diabetes Mellitus in China” and the American Diabetes Association (ADA), prediabetes was diagnosed if any of the following criteria were met (Khan et al., 2019; Chinese Elderly Type 2 Diabetes Prevention and Treatment of Clinical Guidelines Writing Group, 2022): (1) Impaired Fasting Glucose (IFG) (6.1 mmol/L ≤ fasting plasma glucose <7.0 mmol/L, and 2-h post-load plasma glucose <7.8 mmol/L); (2) impaired Glucose Tolerance (IGT) (fasting plasma glucose <6.1 mmol/L, and 7.8 mmol/L ≤ 2-h post-load plasma glucose <11.1 mmol/L); (3) combined IFG and IGT; and (4) glycated hemoglobin (HbA1c) concentration between 5.7% and 6.4%.

#### 2.2.2 Diagnostic criteria for T2DM

The 2020 edition of the “Guidelines for Prevention and Treatment of Type 2 Diabetes Mellitus in China” (Chinese Elderly Type 2 Diabetes Prevention and Treatment of Clinical Guidelines Writing Group, 2022), states that T2DM can be diagnosed if typical symptoms of diabetes (polydipsia, polyphagia, polyuria, and weight loss) are accompanied by any one of the following four criteria: (1) fasting venous plasma glucose ≥7.0 mmol/L; (2) 2-h post-load plasma glucose ≥11.1 mmol/L; (3) Random plasma glucose ≥11.1 mmol/L; (4) HbA1c concentration ≥6.5%.

#### 2.2.3 Diagnostic criteria and staging for DR

The clinical guidelines for diabetic retinopathy published by the American Academy of Ophthalmology and the international clinical staging of diabetic retinopathy provide the following specific staging indications (Wong et al., 2018): DR0 (No Abnormality): No apparent DR; DR1 (Mild Nonproliferative DR [NPDR]): Only microaneurysms are present; DR2 (Moderate Nonproliferative DR): Between mild and severe nonproliferative DR; DR3 (Severe Nonproliferative DR, with any of the following manifestations and no proliferative DR symptoms): (i) > 20 retinal hemorrhages in each of the four quadrants; (ii) definite venous beading in ≥2 quadrants; (iii) significant intraretinal microvascular abnormalities (IRMA) in ≥1 quadrants; DR4 (proliferative DR, with one or both of the following): (i) neovascularization; (ii) vitreous hemorrhage/pre-retinal hemorrhage.

### 2.3 Inclusion criteria

Participants were included in the study if they satisfied the following criteria: (1) a definitive diagnosis of non-diabetes, prediabetes, or T2DM; (2) had undergone fundus photography by experienced ophthalmologists. Those without diabetes or with prediabetes showed no significant retinopathy, whereas patients with T2DM exhibited mild to moderate NPDR changes, as per the DR diagnostic criteria; (3) were aged 18–80 years; and (4) had obtainable basic information and blood sample data.

### 2.4 Exclusion criteria

Participants with the following criteria were excluded if they presented: (1) stage 2.3 hypertension, nephropathy, cardiopathy, systemic lupus erythematosus, or other types of DM besides T2DM, which could possibly affect the ocular fundus vasculature and nerves (diagnosis and staging of hypertension referred to the “2018 Revised Edition of the Chinese Guidelines for Prevention and Treatment of Hypertension” (Joint Committee for Guideline Revision, 2019); (2) history of ocular diseases such as macular degeneration, glaucoma, high myopia, retinal vein occlusion, or retinitis pigmentosa, which affect the ocular fundus vasculature and nerves; (3) diseases severely affecting fundus photography quality, including vitreous opacity, vitreous hemorrhage, or severe cataract; (4) history of ocular trauma or intraocular surgery; (5) abnormal ocular development; (6) severe dyslipidemia, defined as total cholesterol (TC) > 10 mmol/L or triglycerides (TG) > 15 mmol/L; (7) incomplete clinical data or were unable to cooperate with the examination due to poor consciousness.

All study participants satisfied the inclusion criteria.

### 2.5 Research methods

#### 2.5.1 Instrumentation and imaging requirements

Fundus color images were captured using a Canon CR-2 PLUS AF fundus camera in a darkroom by a single operator. For each participant, two 45° images centered on the optic disc were collected for each eye, with the best-quality image from the right eye selected for analysis. The following requirements were adhered to for fundus photography: (1) the fundus color photographs were of sufficient quality to allow identification of 90% of the blood vessels; (2) images were taken at a 45° angle, centered on the optic disc, with clear visibility of major fundus structures such as the optic disc and macula; (3) imaging area was free of shadows or bright reflective areas that could interfere with interpretation; (4) photographs were appropriately exposed, avoiding overexposure or underexposure that could affect image quality; and (5) photographs did not contain obscuring factors such as lens stains, ptosis, excessively long eyelashes, or significant motion artifacts.

#### 2.5.2 Quantitative analysis of vessels in color fundus Photographs

**FIGURE 1:**
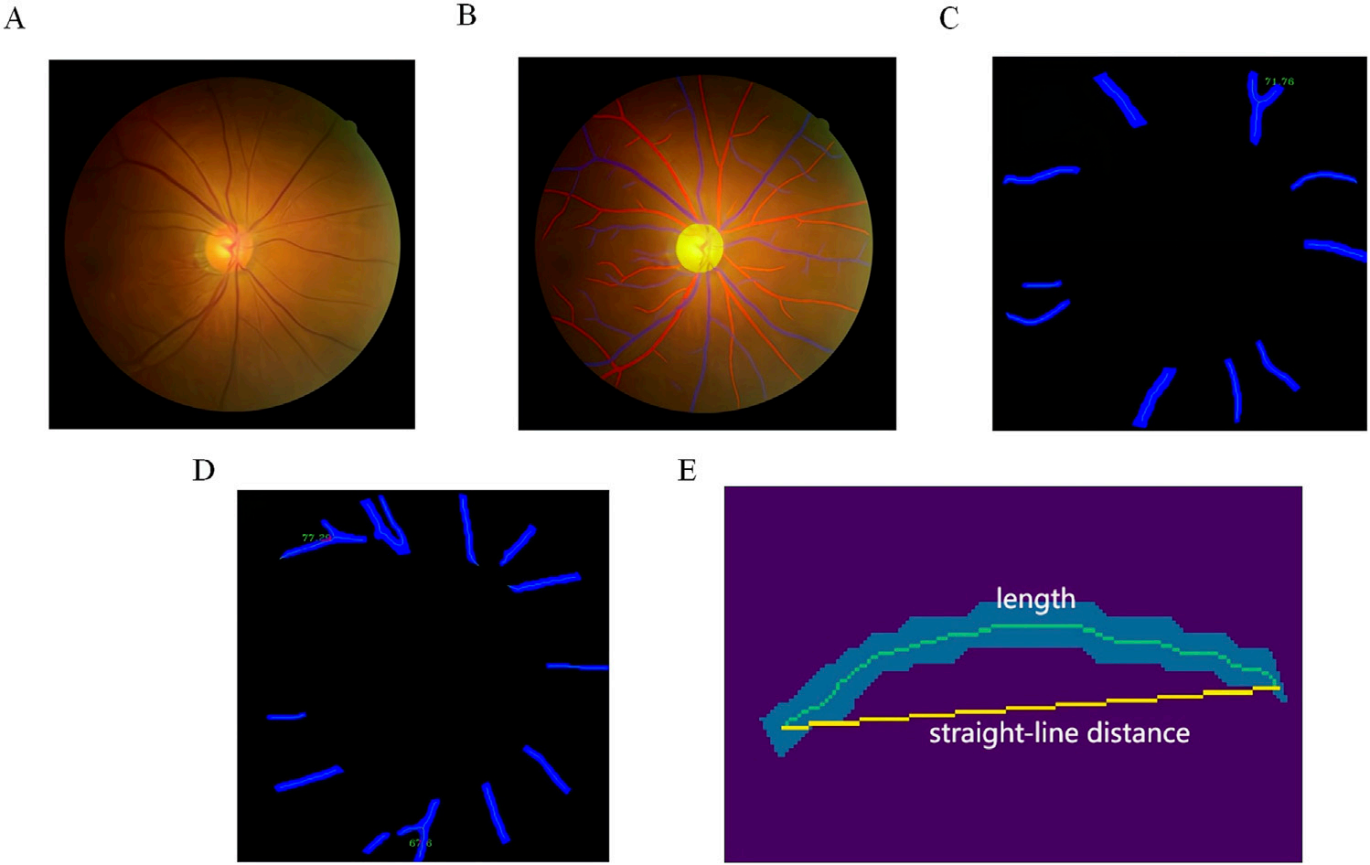
Quantification of Retinal Vascular Parameters from Color Fundus Photography. **(A)** Input fundus image; **(B)** optic disc and artery-vein segmentation, with veins represented in blue, arteries in red, and the optic disc in yellow; **(C)** example of artery bifurcation angle; **(D)** example of vein bifurcation angle; **(E)** example of mean curvature.

We employed an automated measurement system for retinal vascular parameters, which leverages AI technology and was previously developed by our research team (Jiang et al., 2024). This system was used for the segmentation and quantification of color fundus photographs, as depicted in Figure 1. To obtain an accurate measurement of the vessel diameter, we used paired OCT En-face image and color fundus photograph from the same eye of the patient to determine the pixel spacing on the color fundus photograph. The process involved inputting color retinal images and their inter-image spacing to calculate various parameters, including fractal dimension, branch angle-related parameters, length-related parameters, central retinal artery equivalent (CRAE), central retinal vein equivalent (CRVE), and artery-to-vein ratio (AVR). The outcomes were stored in a csv file, with artery-related indicators suffixed with “_a” and vein-related indicators with “_v”.

The segmentation process commenced with the optic disc using HR-Net, a high-resolution semantic feature representation network well-suited for accurate segmentation (Wang et al., 2021). Following the optic disc segmentation, the retinal area was divided into three zones based on the distance from the optic disc: Zone A (0–0.5 disc diameter from the edge), Zone B (0.5–1 disc diameter from the edge), and Zone C (1–2 disc diameters from the edge). We then segmented the retinal artery and vein using HR-Net once more. The resulting masks were skeletonized to extract the centerline of each vessel section, and bifurcation points were identified by applying morphological operations on the extracted centerlines.

##### 2.5.2.1 Fractal dimensions

We analyzed four distinct fractal dimensions of arteries and veins: capacity dimension (D0), entropy dimension (D1), correlation dimension (D2), and singularity length (SL). Higher values of fractal dimensions indicated more complex vascular patterns, and *vice versa*. These dimensions were calculated using the centerlines extracted in the previous step as input for the box-counting method implemented in the PVBM package (Fhima et al., 2023).

##### 2.5.2.2 Branching angle

For arteries and veins in Zone C, we calculated the average branch angle (angle_avg), average branch asymmetry (asymmetry_avg), and average number of branches (branch_avg), following the definitions provided by Sun H. et al. (2022). Where no branches were present in Zone C, the corresponding entries in the table were left blank.

##### 2.5.2.3 Tortuosity

We calculated additional parameters for arteries and veins in Zone C, including the average length (length_avg), average tortuosity (curvature_avg), overall length density (vessel_length_density), and vessel area density (vessel_density). Average tortuosity is defined as the ratio of the actual integral length of the vessel segment centerline to the length of a straight line connecting the endpoints of the vessel segment, with lower values indicating a straighter vessel.

##### 2.5.2.4 CRAE, CRVE, AVR

Based on the Knudston formula (Knudtson et al., 2003), we calculated the CRAE, the CRVE, and the AVR, as detailed below:

$$W_{artery}=0.88\sqrt{W_{1}^{2}+W_{1}^{2}};\;W_{vein}=0.95\sqrt{W_{1}^{2}+W_{1}^{2}}$$

where W_1_ and W_2_ represent the narrower and wider retinal branches.

### 2.6 Statistical methods

Categorical variables were expressed as percentages, and continuous variables were presented as mean ± standard deviation (MD ± SD) for those with a normal distribution, or as median P50 (P25, P75) for those without. To compare means across multiple groups that met the criteria for normal distribution and homogeneity of variance, One-way ANOVA was utilized. The Least Significant Difference (LSD) test was applied for pairwise comparisons among these groups. For two-group comparisons with non-normal distributions, the Mann–Whitney *U* test was employed, and for multiple-group comparisons in such cases, the Kruskal–Wallis H test was used. The significance level was set at α = 0.05, with *P* < 0.05 indicating a statistically significant difference. Variables with P-values <0.05 in group comparisons were subjected to binary logistic regression analysis to identify independent risk factors for T2DM with DR. Receiver operating characteristic (ROC) curves were plotted for the subjects, and the AUC, sensitivity, and specificity of each parameter were determined.

### 2.7 Study flowcharts

**FIGURE 2:**
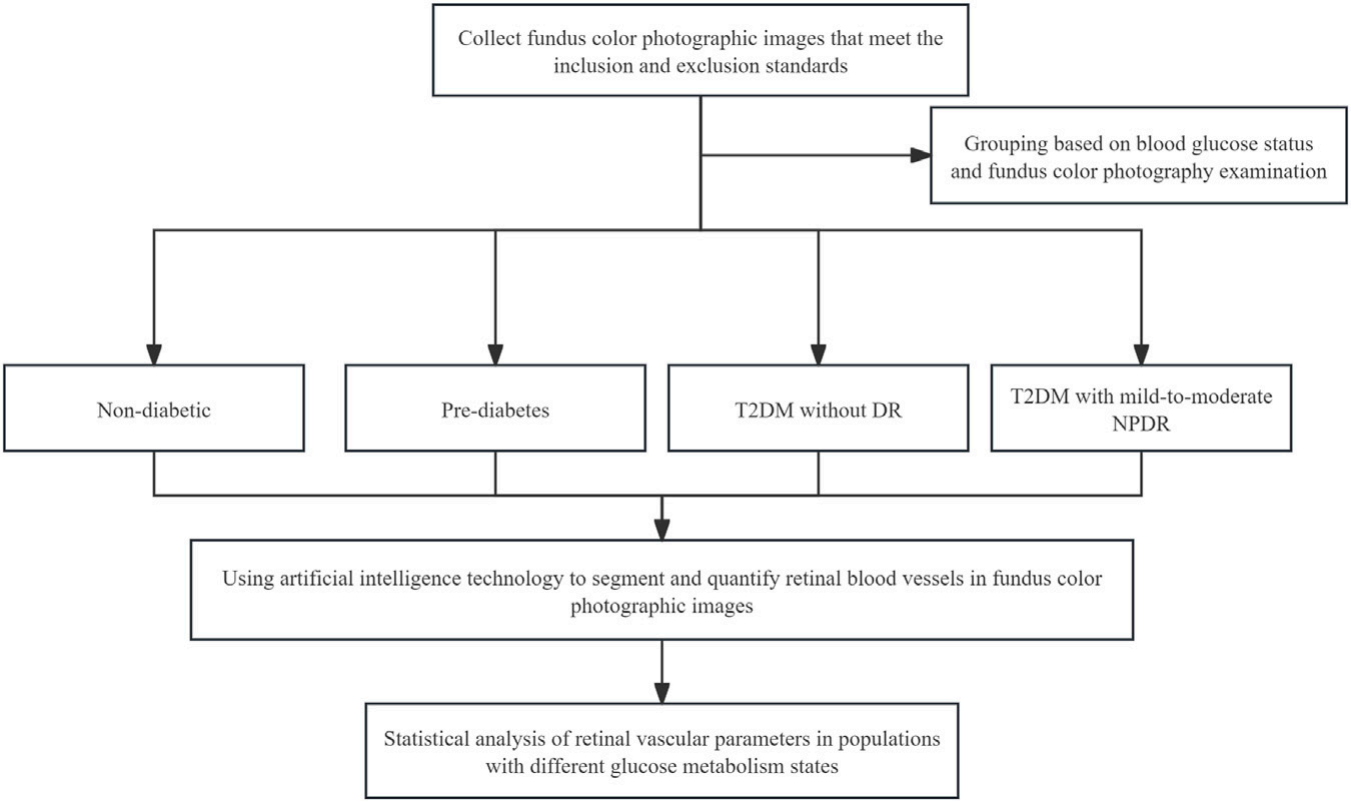
Study flowchart.

The study flowchart is shown in Figure 2.

## 3 Results

### 3.1 General characteristics

**FIGURE 3:**
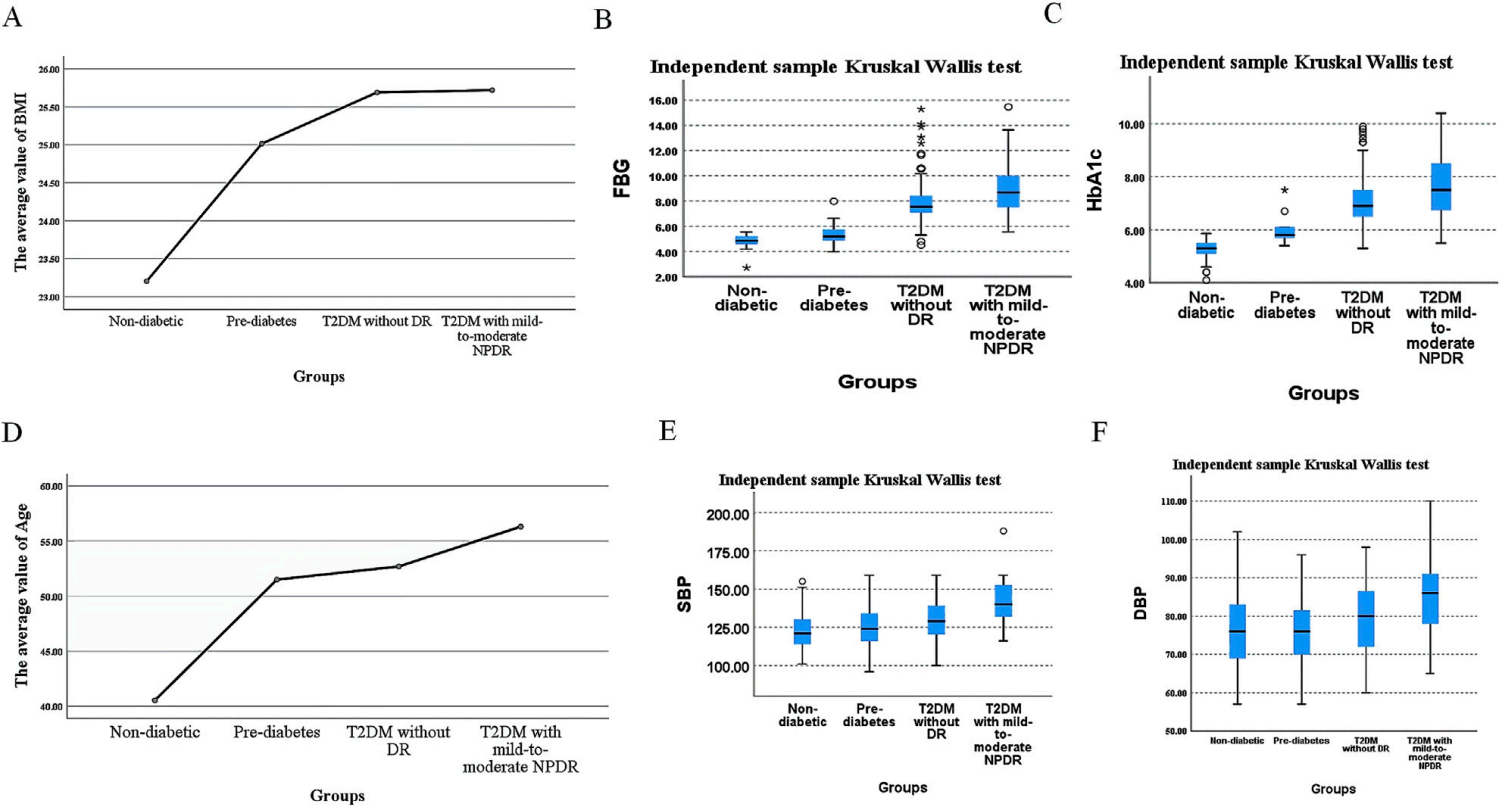
Differences in BMI, FBG, HbA1c, Age, SBP, and DBP Values Across Different Groups. **(A)**. Mean BMI, **(B)** median FBG, **(C)** median HbA1c, **(D)** mean age, **(E)** median SBP, and **(F)** median DBP values across different groups.

Chi-square testing revealed no significant difference in sex distribution among the four groups, with the results being statistically insignificant (*P* > 0.05), as detailed in Table 1. The values of the body mass index (BMI), FBG, HbA1c, age, SBP, and DBP showed an upward trend with the severity of glycemic abnormalities and the occurrence of mild-to-moderate NPDR. In pairwise comparisons among individuals with pre-diabetes, T2DM without DR, and T2DM with mild-to-moderate NPDR, BMI values did not differ significantly (*P* > 0.05). In contrast, significant differences were found in FBG, HbA1c, SBP, and DBP levels among these groups (*P* < 0.05). Within the T2DM patient population, FBG, HbA1c, age, SBP, and DBP were identified as statistically significant predictors for the occurrence of mild-to-moderate NPDR (*P* < 0.05). The incidence of mild-to-moderate NPDR was associated with elevated levels of FBG, HbA1c, age, SBP, and DBP, as further illustrated in Table 2 and Figure 3.

**TABLE 1:** Comparison of demographic and physiological characteristics differences between study groups.

Variable	Meaning of variables	Non-diabetic (n = 54)	Pre-diabetes (n = 71)	T2DM without DR (n = 144)	T2DM with mild-to-moderate NPDR (n = 51)	F/H/χ^2^	P Value
Male	Male	30 (55.6%)	39 (54.9%)	94 (65.3%)	39 (76.5%)	7.567	0.056
Age*	Age	40.54 ± 8.61	51.51 ± 7.60	52.70 ± 7.87	56.31 ± 6.79	42.827	0.000
BMI(kg/m^2^)*	Body mass index	23.20 ± 3.47	25.02 ± 2.90	25.69 ± 2.97	25.72 ± 3.09	9.398	0.000
SBP(mmHg)	Systolic blood pressure	121 (114,130.5)	124 (116,134)	129 (120,139)	140 (132,153)	47.398	0.000
DBP(mmHg)	Diastolic blood pressure	76 (68.75,83.06)	76 (70,82)	80 (72,86.75)	86 (78,91)	26.999	0.000
FBG (mmol/L)	Fasting blood glucose	4.87 (4.6,5.2)	5.2 (4.8,5.73)	7.55 (7.09,8.42)	8.67 (7.51,10.03)	218.686	0.000
HbA1c (%)	Glycated hemoglobin	5.3 (5.1,5.5)	5.8 (5.7,6.1)	6.9 (6.5,7.5)	7.5 (6.7,8.5)	215.963	0.002

*Conforms to the requirements of normal distribution and homogeneity of variance.

**TABLE 2:** Pairwise comparison of BMI, FBG, HbA1c, age, SBP, and DBP values across different groups.

Groups	Groups	P value BMI	P value FBG	P value HbA1c	P value Age	P value SBP	P value DBP
Non-diabetic	Pre-diabetes	0.001	0.065	0.000	0.000	0.384	0.694
	T2DM without DR	0.000	0.000	0.000	0.000	0.002	0.055
	T2DM with mild-to-moderate NPDR	0.000	0.000	0.000	0.000	0.000	0.000
Pre-diabetes	non-diabetic	0.001	0.065	0.000	0.000	0.384	0.694
	T2DM without DR	0.129	0.000	0.000	0.291	0.019	0.009
	T2DM with mild-to-moderate NPDR	0.211	0.000	0.000	0.000	0.000	0.000
T2DM without DR	non-diabetic	0.000	0.000	0.000	0.000	0.002	0.055
	Pre-diabetes	0.129	0.000	0.000	0.291	0.019	0.009
	T2DM with mild-to-moderate NPDR	0.952	0.025	0.034	0.005	0.000	0.002
T2DM with mild-to-moderate NPDR	non-diabetic	0.000	0.000	0.000	0.000	0.000	0.000
	Pre-diabetes	0.211	0.000	0.000	0.001	0.000	0.000
	T2DM without DR	0.952	0.025	0.034	0.005	0.000	0.002

### 3.2 Comparison of retinal vascular parameters

Our study identified significant differences in retinal vascular parameters, including d0_v, d1_v, d2_v, sl_v, length_avg_a, length_avg_v, branch_avg_v, angle_avg_v, asymmetry_avg_v, curvature_avg_v, vessel_length_density, and vessel_density, among various groups as detailed in Table 3. Among these parameters, upon pairwise comparisons for variables showing significant inter-group differences, d0_v, d1_v, d2_v, sl_v, and curvature_avg_v did not exhibit any significant different in relation to blood glucose variations or the occurrence of mild-to-moderate NPDR (*P* > 0.05), as indicated in Table 4.

**TABLE 3:** Comparison of differences in retinal vascular parameters between study groups.

Variable	Meaning of variables	Non-diabetic (n = 54)	Pre-diabetes (n = 71)	T2DM without DR (n = 144)	T2DM with mild-to-moderate NPDR (n = 51)	F/H/χ^2^	P Value
AVR※	Artery-to-vein ratio	0.73 ± 0.07	0.74 ± 0.07	0.73 ± 0.06	0.72 ± 0.05	1.085	0.356
CRAE※	Central retinal artery equivalent	186.97 ± 11.87	187.55 ± 12.45	189.45 ± 11.84	188.87 ± 11.60	0.765	0.514
CRVE※	Central retinal vein equivalent	256.96 ± 20.66	255.23 ± 23.55	261.29 ± 19.84	263.83 ± 23.50	2.218	0.086
d0_a※	Arterial entropy dimension	1.16 ± 0.03	1.16 ± 0.04	1.16 ± 0.03	1.16 ± 0.04	0.196	0.899
d1_a※	Arterial entropy dimension	1.11 ± 0.03	1.11 ± 0.04	1.11 ± 0.04	1.11 ± 0.04	0.332	0.802
d2_a※	Arterial correlation dimension	1.09 ± 0.04	1.09 ± 0.04	1.08 ± 0.04	1.08 ± 0.04	0.452	0.716
sl_a	Arterial singularity length	1.01 (0.92,1.09)	1.03 (0.95,1.08)	1.02 (0.94,1.10)	1.05 (0.96,1.13)	2.854	0.415
d0_v	Venous entropy dimension	1.17 (1.14,1.19)	1.14 (1.12,1.17)	1.15 (1.12,1.18)	1.13 (1.12,1.16)	17.177	0.001
d1_v	Venous entropy dimension	1.12 (1.09,1.13)	1.09 (1.06,1.12)	1.10 (1.06,1.12)	1.09 (1.07,1.11)	12.772	0.005
d2_v	Venous correlation dimension	1.10 (1.07,1.11)	1.07 (1.04,1.09)	1.08 (1.04,1.10)	1.07 (1.04,1.09)	10.914	0.012
sl_v*	Venous singularity length	1.05266 ± 0.10106	1.00335 ± 0.13937	1.00883 ± 0.12278	0.98112 ± 0.13042	3.092	0.027
length_avg_a	Average length of arteries	127.37 (114.42,140.81)	131.86 (122.04,147.02)	135.30 (122.70.150.95)	142.51 (130.78,157.91)	19.267	0.000
curvature_avg_a	Average arterial tortuosity	1.100 (1.097,1.110)	1.100 (1.094,1.105)	1.100 (1.093,1.104)	1.099 (1.099,1.102)	6.347	0.096
angle_avg_a*	Average arterial branching angle	87.71 ± 13.76	88.65 ± 15.04	89.43 ± 14.85	84.95 ± 12.10	1.277	0.282
asymmetry_avg_a*	Average arterial branching asymmetry	44.47 ± 12.78	44.94 ± 9.81	44.34 ± 11.58	43.07 ± 11.95	0.273	0.845
branch_avg_a	Average arterial branching number	3.67 (3.00,4.40)	3.33 (2.80,4.29)	3.43 (2.76,4.00)	3.20 (2.67,3.71)	6.212	0.102
length_avg_v*	Average length of veins	135.37 ± 20.07	140.79 ± 20.92	144.63 ± 21.40	151.63 ± 22.17	5.665	0.001
curvature_avg_v	Average venous tortuosity	1.101 (1.094,1.110)	1.096 (1.902,1.102)	1.094 (1.088,1.102)	1.093 (1.085,1.098)	15.017	0.002
angle_avg_v*	Average venous branching angle	76.36 ± 10.65	78.57 ± 13.62	76.70 ± 11.52	70.28 ± 15.05	4.715	0.003
asymmetry_avg_v*	Average venous branching asymmetry	38.43 ± 10.50	36.33 ± 11.23	37.83 ± 11.03	31.89 ± 11.51	4.172	0.006
branch_avg_v	Average venous branching number	3.00 (2.50,3.50)	3.00 (2.50,3.68)	2.80 (2.40,3.33)	2.67 (2.00,3.00)	17.758	0.000
vessel_length_density*	Overall length density	0.00805 ± 0.00127	0.00692 ± 0.00123	0.00705 ± 0.00125	0.00627 ± 0.00133	17.870	0.000
vessel_density*	Vessel area density	0.13149 ± 0.01898	0.11560 ± 0.02049	0.11803 ± 0.01944	0.10686 ± 0.02077	14.060	0.000

*Conforms to the requirements of normal distribution and homogeneity of variance.

**TABLE 4:** Pairwise Comparison of d0_v, d1_v, d2_v, sl_v, and curvature_avg_v Values Across Different Groups.

Groups 1	Groups 2	P Value d0_v	P Value d1_v	P Value d2_v	P Value sl_v	P Value curvature_avg_v
Non-diabetic	Pre-diabetes	0.001	0.002	0.003	0.029	0.012
	T2DM without DR	0.004	0.015	0.028	0.028	0.002
	T2DM with mild-to-moderate NPDR	0.001	0.002	0.005	0.004	0.001
Pre-diabetes	Non-diabetic	0.001	0.002	0.003	0.029	0.012
	T2DM without DR	0.234	0.208	0.205	0.762	0.806
	T2DM with mild-to-moderate NPDR	0.574	0.879	0.943	0.332	0.139
T2DM without DR	Non-diabetic	0.004	0.015	0.028	0.028	0.002
	Pre-diabetes	0.234	0.208	0.205	0.762	0.806
	T2DM with mild-to-moderate NPDR	0.091	0.196	0.227	0.173	0.147
T2DM with mild-to-moderate NPDR	Bon-diabetic	0.001	0.002	0.005	0.004	0.001
	Pre-diabetes	0.574	0.879	0.943	0.332	0.139
	T2DM without DR	0.091	0.196	0.227	0.173	0.147

**FIGURE 4:**
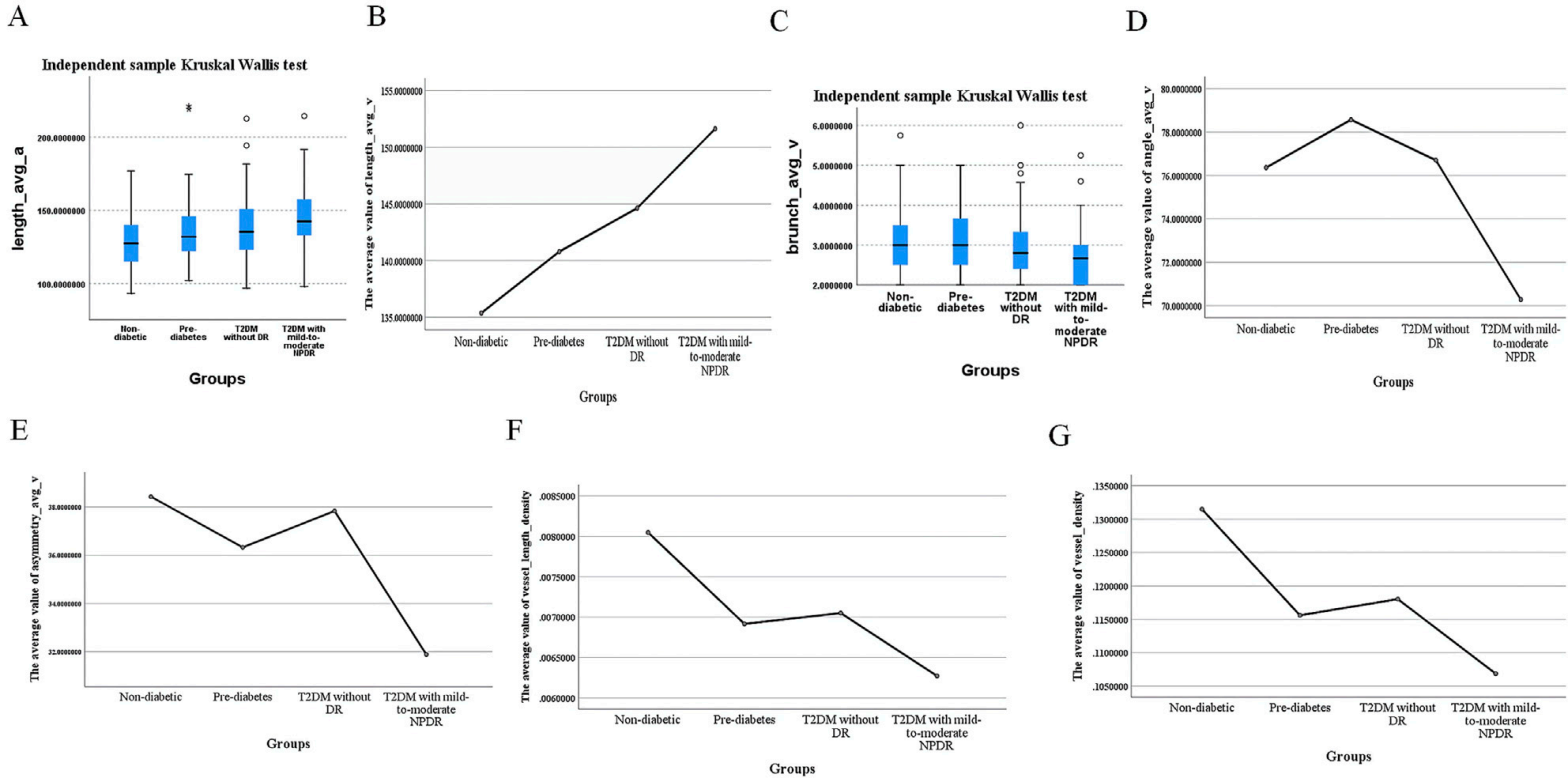
Differences in length_avg_a, length_avg_v, branch_avg_v, angle_avg_v, asymmetry_avg_v, vessel_length_density, and vessel_density Values Across Different Groups. **(A)** Median values of length_avg_a, **(B)** mean values of length_avg_v, **(C)** median values of branch_avg_v, **(D)** mean values of angle_avg_v, **(E)** mean values of asymmetry_avg_v, **(F)** mean values of vessel_length_density, **(G)** mean values of vessel_density in different groups.

A notable exception was branch_avg_v, which showed significant differences in pairwise comparisons among individuals with pre-diabetes, T2DM without DR, and T2DM with mild-to-moderate NPDR (*P* < 0.05). Branch_avg_v demonstrated a decreasing trend with the worsening of glycemic abnormalities and the occurrence of mild-to-moderate NPDR. In patients with T2DM with mild-to-moderate NPDR, we observed significant increases in length_avg_a and length_avg_v, along with significant decreases in branch_avg_v, angle_avg_v, asymmetry_avg_v, vessel_length_density, and vessel_density (*P* < 0.05, for all), as presented in Table 5 and Figure 4.

**TABLE 5:** Pairwise Comparison of length_avg_a, length_avg_v, branch_avg_v, angle_avg_v, asymmetry_avg_v, vessel_length_density, and vessel_density Values Across Different Groups.

Groups 1	Groups 2	P value length_avg_a	P value length_avg_v	P value branch_avg_v	P value angle_avg_v	P value asymmetry_avg_v	P value vessel_length_density	P value vessel_density
Non-diabetic	Pre-diabetes	0.070	0.158	0.639	0.330	0.296	0.000	0.000
	T2DM without DR	0.005	0.007	0.118	0.865	0.737	0.000	0.000
	T2DM with mild-to-moderate NPDR	0.000	0.000	0.000	0.013	0.003	0.000	0.000
Pre-diabetes	Non-diabetic	0.070	0.158	0.639	0.330	0.296	0.000	0.000
	T2DM without DR	0.401	0.212	0.022	0.306	0.352	0.466	0.399
	T2DM with mild-to-moderate NPDR	0.005	0.006	0.000	0.000	0.030	0.006	0.017
T2DM without DR	Non-diabetic	0.005	0.007	0.118	0.865	0.737	0.000	0.000
	Pre-diabetes	0.401	0.212	0.022	0.306	0.352	0.466	0.399
	T2DM with mild-to-moderate NPDR	0.016	0.044	0.019	0.002	0.001	0.000	0.001
T2DM with mild-to-moderate NPDR	Non-diabetic	0.000	0.000	0.001	0.013	0.003	0.000	0.000
	Pre-diabetes	0.005	0.006	0.001	0.000	0.030	0.006	0.017
	T2DM without DR	0.016	0.044	0.019	0.002	0.001	0.000	0.001

### 3.3 Logistic regression analysis of retinal vascular parameters in patients with T2DM

To address the potential interactions among retinal vascular parameters, we conducted a binary multivariate logistic regression analysis including only the parameters that were statistically significant from the intergroup comparisons of patients with T2DM, as detailed in Table 5. The analysis revealed that length_avg_a, branch_avg_v, angle_avg_v, asymmetry_avg_v, vessel_length_density, and vessel_density were independently associated with the occurrence of mild-to-moderate NPDR (Table 6).

**TABLE 6:** Logistic regression analysis of retinal vascular parameters in patients with mild-to-moderate NPDR.

Variable	β	Wald χ2	SE	P value	OR	95% CI
length_avg_a	0.019	6.177	0.008	0.013	1.019	1.004–1.035
length_avg_v	0.015	3.844	0.008	0.05	1.015	1.00–1.03
branch_avg_v	−0.566	4.429	0.269	0.035	0.568	0.335–0.692
angle_avg_v	−0.042	9.030	0.014	0.003	0.959	0.933–0.985
asymmetry_avg_v	−0.048	9.640	0.015	0.002	0.953	0.925–0.982
vessel_length_density	−470.729	12.142	135.089	0.000	6.25 × 10^−4^	4.79 × 10^-4^–8.14 × 10^−4^
vessel_density	−25.579	10.608	8.467	0.001	9.73 × 10^−4^	9.57 × 10^-4^–9.89 × 10^−4^

### 3.4 Diagnostic efficacy of vascular parameters for mild-to-moderate NPDR

**FIGURE 5:**
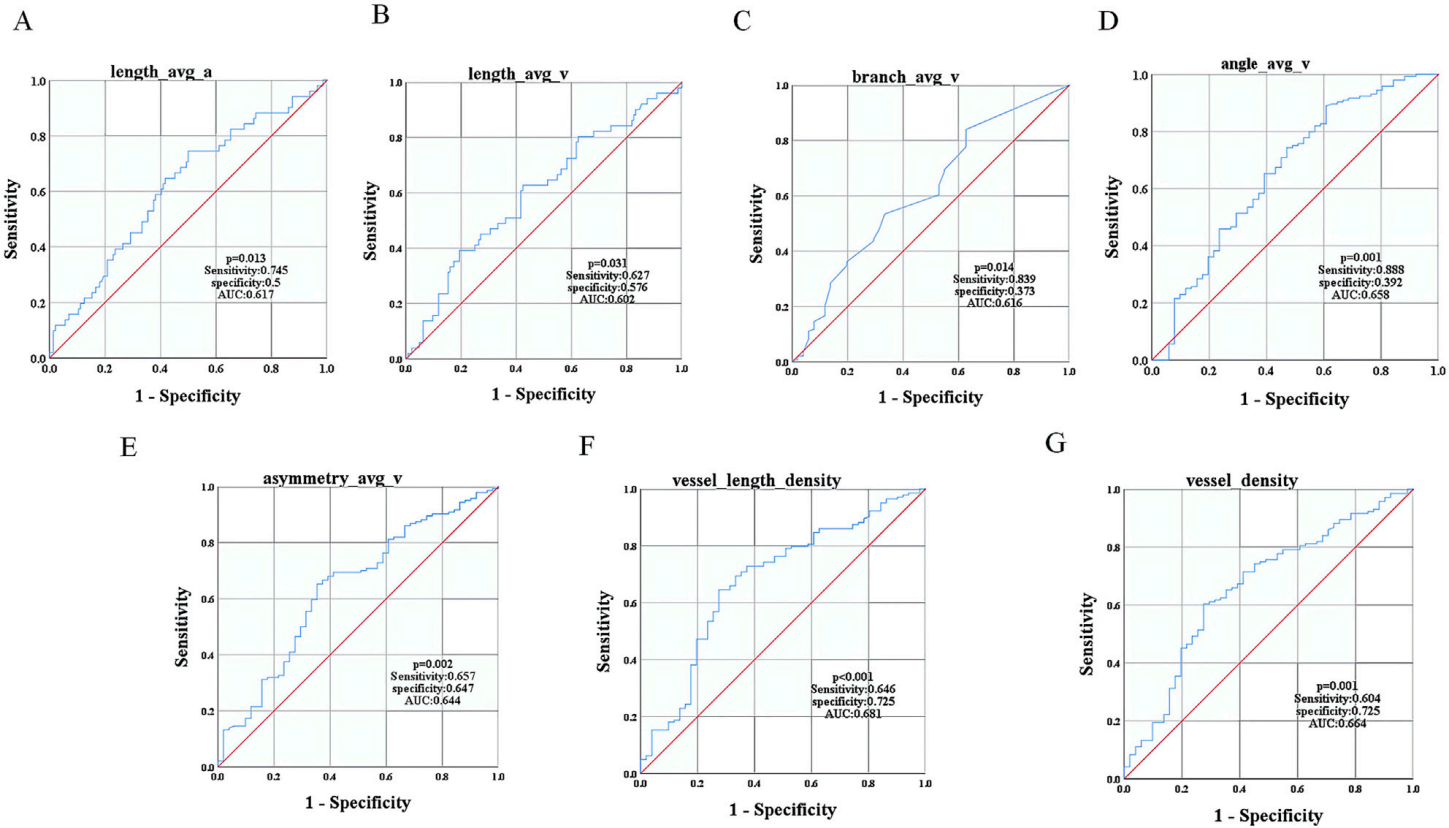
ROC Curve Analysis of Vascular Parameters in Mild-to-Moderate NPDR Patients. ROC curves for **(A)** length_avg_a, **(B)** length_avg_v, **(C)** branch_avg_v, **(D)** angle_avg_v, **(E)** asymmetry_avg_v, **(F)** vessel_length_density, and **(G)** vessel_density.

Patients with T2DM and mild-to-moderate NPDR showed significant differences (*P* < 0.05) in the retinal vascular parameters length_avg_a, length_avg_v, branch_avg_v, angle_avg_v, asymmetry_avg_v, vessel_length_density, and vessel_density when compared with the T2DM group without DR (Table 5). We selected these seven indices as test variables, with the presence of mild-to-moderate NPDR serving as the state variable. To evaluate the diagnostic efficacy of these indices for mild-to-moderate NPDR, ROC curves were plotted for each. The ROC curve analysis confirmed that these parameters had diagnostic value for mild-to-moderate NPDR (*P* < 0.05), with AUCs of 0.617, 0.602, 0.616, 0.658, 0.644, 0.681, and 0.664, respectively. The corresponding sensitivities were 0.745, 0.627, 0.839, 0.888, 0.657, 0.646, and 0.604, and the specificities were 0.5, 0.576, 0.373, 0.392, 0.647, 0.725, and 0.725, respectively (Figure 5).

## 4 Discussion

DR, a major ocular complication of diabetes, poses a significant threat to global ocular health (Wiedemann, 2023). The global burden of DR is not only increasing but also shifting geographically, moving from middle-to high-income countries toward low- and middle-income regions, which further exacerbates the challenges faced by global health systems (Tan and Wong, 2022). The retinal microvascular system is essential for the visual function of the neuroretina and maintains the homeostatic environment of the retina (Huang, 2020). Under normal physiological conditions, protective factors such as antioxidant enzymes and insulin effectively protect blood vessels from damage. However, hyperglycemic conditions inhibit these protective factors, leading to vascular injury (Wright et al., 2020). For patients with diabetes, changes in the retinal microvasculature often signal the onset of early DR. The early clinical features of DR include subtle and complex vascular wall changes, such as pericyte loss, and microvascular damage caused by chronic hyperglycemia. These changes are important early warning signals for disease progression but are often difficult to detect directly by visual inspection (Zhang et al., 2021). Therefore, an in-depth investigation of vascular parameter changes in DR and accurate prediction of its development trends are crucial for early detection, timely intervention, and effective treatment of the disease. Color fundus photography, a commonly used clinical examination method, enables systematic analysis of subtle changes in retinal blood vessels and aids in detecting signs of DR at the preclinical stage, facilitating early intervention. By monitoring these subtle changes in microvascular structure, we can obtain valuable diagnostic evidence, advance the timing of treatment, and take effective measures to block or delay the progression of the disease before DR leads to irreversible visual impairment.

In this study, we conducted a quantitative analysis of retinal vasculature among individuals with varying status of glucose metabolism: patients without diabetes, pre-diabetes, T2DM without DR, and T2DM with mild-to-moderate NPDR. Our findings indicated that the branch_avg_v value was statistically significant when comparing any two groups among pre-diabetes, T2DM without DR, and T2DM with mild-to-moderate NPDR, suggesting its potential as an early warning indicator for glucose metabolism abnormalities and the progression of ocular fundus lesions.

In patients with T2DM with mild-to-moderate NPDR, we observed increased length_avg_a and length_avg_v values, along with decreased branch_avg_v, angle_avg_v, asymmetry_avg_v, vessel_length_density, and vessel_density; these differences were statistically significant. Notably, length_avg_a, angle_avg_v, branch_avg_v, asymmetry_avg_v, vessel_length_density, and vessel_density were independently associated with the occurrence of mild-to-moderate NPDR. The study highlighted that initial retinal vascular changes were particularly prominent in retinal veins, primarily due to their unique anatomical characteristics, such as relatively larger lumen diameters, weak vessel wall structures, and the absence of a fully developed smooth muscle layer. These features make the retinal venous system more sensitive to hemodynamic fluctuations and local tissue damage, and suggest that retinal veins can serve as biomarkers for early pathological changes (Moss, 2021).

The increase in both length_avg_a and length_avg_v values was closely associated with a significant elevation in vascular tortuosity, suggesting that as vascular tortuosity intensifies, the overall average length of the vascular system correspondingly increases. Studies have highlighted the complexity of vascular pathological changes during the development of DR, originating from damage to microvascular endothelial cells and pericytes. This cellular-level injury destabilizes the vascular wall, rendering the vascular structure more susceptible to hemodynamic fluctuations, thereby inducing vascular tortuosity. Lee et al. (2018) conducted an in-depth analysis and noted a marked increase in vascular tortuosity among patients with mild-to-moderate NPDR compared to those without DR. Similarly, Forster et al. (2021) determined that vascular tortuosity increased as patients with T2DM progressed from no DR to mild-to-moderate NPDR. Consequently, vascular tortuosity has emerged as a prominent hallmark in the pathological progression of DR and is a direct manifestation of microvascular dysfunction and hemodynamic disturbance. However, in our study, differences in vascular tortuosity of patients with T2DM without DR and those with mild-to-moderate NPDR did not reach statistical significance. This may be due to variations in sample size, research methods, or subtle differences in disease staging, or it could be that at this specific stage, changes in vascular tortuosity have not yet reached a level sufficient to form a significant difference. Nevertheless, these findings still provide new insights into the pathophysiological processes of DR and offer valuable references for future research.

In populations of patients with T2DM without DR and those with mild-to-moderate NPDR, a decline in both angle_avg_v and asymmetry_avg_v values was observed. This alteration may be attributed to adverse effects of chronic hyperglycemia on the retinal vasculature. Specifically, a hyperglycemic environment promotes the reaction between glucose and vascular wall components, leading to the formation of advanced glycosylation end products (AGEs) (Auvazian et al., 2021). The gradual accumulation of AGEs results in the thickening and stiffening of the vascular wall. This process not only alters the structure of the blood vessels but also impairs their function, particularly reducing the elasticity at vascular bifurcations. As a result, the branching angles of the retinal vessels decrease, and asymmetry increases as the pathology progresses, changes that typically signify microvascular damage (Cabrera DeBuc et al., 2018).

The present study revealed a declining trend in the branch_avg_v value among the three study populations: individuals with pre-diabetes, T2DM without DR, and T2DM with mild-to-moderate NPDR, as hyperglycemia worsened and mild-to-moderate NPDR developed. This change was statistically significant in pairwise comparisons across the three populations. The downward trend was closely associated with the hyperglycemic status of the patient. In a hyperglycemic environment, the normal glycolytic process is impeded, potentially triggering reactions such as the activation of the sorbitol synthesis pathway. This leads to increased intracellular osmolarity, electrolyte imbalance, and metabolic disturbances (Singh et al., 2021). These pathophysiological changes not only affect the normal structure and function of retinal vessels but may also interfere with vascular branching and developmental processes. Moreover, hyperglycemia can inhibit the uptake and synthesis of inositol by pericytes, inducing abnormal inositol phospholipid metabolism. This further impairs cellular proliferative capacity and vitality, adversely affecting the formation and branching of retinal vessels. Severe hyperglycemia readily induces apoptosis of pericytes in retinal vessels, resulting in a further reduction in vessel number and branching (Yang and Liu, 2022). Therefore, the branch_avg_v value serves as an early warning indicator of the metabolic state of glucose and the progression of lesions in the ocular fundus. By observing and monitoring changes in the branch_avg_v value, we can identify the risk of abnormal glucose metabolism and ocular fundus lesions earlier, enabling timely interventions to delay disease progression and improve patient prognosis.

In the assessment of visual function, vessel length density and vessel density have been established as effective indicators (Waheed et al., 2023). Vessel density is commonly regarded as the counterpart of vessel length density (Sun et al., 2021). The retinal vasculature is a dynamic and sensitive system that undergoes corresponding alterations in response to pathological changes within the retina. Decreases in vessel density or vessel length density often signal the occurrence of vascular occlusion and loss, and simultaneously reflect a decline in perfusion levels within inner retinal tissue structures (You et al., 2019). For microvascular diseases, particularly ischemic complications such as diabetic macular ischemia, the measurement of vessel density is crucial for gaining deeper insights into disease progression and pathological alterations (Pramil et al., 2021). Vessel density and vessel length density are sensitive biomarkers not only for the early detection of DR (Alam et al., 2020), but also for evaluating therapeutic efficacy and predicting the trend in disease progression. Notably, vessel length density and vessel density are typically difficult to observe directly with the naked eye, necessitating the utilization of advanced technological instruments, including AI-assisted vascular parameter measurement techniques, to accurately and efficiently acquire these critical pieces of information. Through this approach, subtle changes within the retinal vasculature can be detected early, providing precious time for disease prevention and treatment.

This study investigates the variations in vascular parameters, as revealed by color fundus photography in patients classified into four categories: patients without diabetes, pre-diabetes, T2DM without DR, and T2DM with mild-to-moderate NPDR. Notably this study showed that as glucose metabolism became abnormal and pancreatic islet function declined in patients without diabetes to pre-diabetes and further to T2DM without DR, the branch_avg_v values did not differ significantly between the patients without diabetes and pre-diabetes groups. However, a declining pattern in branch_avg_v values was observed among pre-diabetes, T2DM without DR, and T2DM with mild-to-moderate NPDR populations as glucose abnormalities intensified and mild-to-moderate NPDR developed, with statistically significant differences in pairwise comparisons. This indicates that the branch_avg_v value can serve as a detection and monitoring indicator for these three distinct populations, aiding in the early identification of abnormal glucose metabolism and the risk of ocular fundus pathology.

Focusing the analysis on the T2DM population, significant changes were observed in vascular parameters including length_avg_a, length_avg_v, branch_avg_v, angle_avg_v, asymmetry_avg_v, vessel_length_density, and vessel_density. These changes closely correlated to the occurrence of mild-to-moderate NPDR. This discovery underscores the pivotal role of morphological changes in ocular fundus vasculature acting as an early warning of DR among patients with T2DM and provides a non-invasive and highly accessible screening method for clinical practice. Color fundus photography, with its widespread availability and efficacy, offers the potential for large-scale screening for DR. By regularly monitoring changes in the aforementioned vascular parameters, we can identify high-risk individuals for DR among patients with T2DM early on, allowing for intervention measures to be applied before irreversible damage occurs, thereby achieving effective prevention and treatment of DR.

The present study examined the trends in BMI, FBG, HbA1c, age, SBP, and DBP levels in patients with different glucose metabolism and retinal statuses, with a particular focus on their associations with the occurrence of mild-to-moderate NPDR. The results indicated that the BMI values were not significantly different in pairwise comparisons among individuals with pre-diabetes, T2DM without DR, and T2DM with mild-to-moderate NPDR. In contrast, FBG, HbA1c, SBP, and DBP values were significantly different in the pairwise comparisons across these groups. Elevated levels of FBG, HbA1c, SBP, and DBP served as warning indicators for abnormal glucose metabolism and the development of DR. BMI, FBG, HbA1c, age, SBP, and DBP values exhibited an upward pattern as glycemic disturbances intensified and mild-to-moderate NPDR developed.

In a detailed analysis of patients with T2DM, this study found that an increase in BMI was not significantly associated with the occurrence of mild-to-moderate NPDR, suggesting that an elevated BMI alone is not sufficient to predict the development of mild-to-moderate NPDR. However, previous studies have pointed out that an increased BMI can accelerate the overall progression of DR (Song et al., 2019; Gong et al., 2023), a conclusion that aligns with the trends observed in this study. Among patients with T2DM, elevated FBG, increased HbA1c levels, advanced age, higher SBP, and elevated DBP were statistically significant predictors of mild-to-moderate NPDR, findings that are consistent with previous research (Song et al., 2019; Lin et al., 2021; Gong et al., 2023; Hou et al., 2023). Although advancing age is inevitable, the control of the glycemic status and blood pressure levels is within our capacity. The importance of glycemic control has always been recognized, but the crucial role of blood pressure control is sometimes overlooked. Effective blood pressure management can improve endothelial function, enhance capillary dilation and microvascular perfusion, which not only facilitates the delivery of insulin to muscle tissue but also maintains interstitial insulin at appropriate levels (Zhang et al., 2020). These physiological effects can effectively inhibit the occurrence and progression of DR (Fung et al., 2022).

During follow-up of fundus color photography images in patients with T2DM, heightened attention must be given to specific vascular parameter changes: an increase in the length_avg_a and length_avg_v values, and decreases in the branch_avg_v, angle_avg_v, asymmetry_avg_v, vessel_length_density, and vessel_density. This study showed that these parameters indicate the need for further control of body weight, FBG, HbA1c levels, SBP, and DBP in this patient population. To more effectively manage the disease, it is essential to reduce the relevant targeted goal values and intensify the monitoring of vascular parameter changes in fundus color photography images. This can be achieved by shortening the observation interval, which allows for more timely adjustments to the treatment regimen, ensuring prompt and effective monitoring and management of the ocular health status of patients with diabetes.

This study not only deepens our understanding of the pathogenesis of diabetic microvascular complications but also offers scientific support for the formulation of comprehensive management strategies for diabetes and its complications. It holds the potential to drive innovations in DR prevention and treatment technologies, enhance patients’ quality of life, and reduce the societal medical burden, demonstrating significant clinical and public health value.

This study primarily explores the potential associations between early stages of DR, particularly mild-to-moderate NPDR, and retinal vascular parameters. Despite certain advances, the study has some limitations. First, the study did not include vascular parameters from other stages of DR. Future studies should incorporate patients with varying severities of DR to comprehensively assess the impact of metabolic indicator changes on DR progression. Secondly, although data from 320 individuals were collected, the sample size may be have been insufficient for the analysis of specific subgroups, such as the non-diabetic group and the T2DM with mild-to-moderate NPDR, which ultimately affected the robustness of the statistical results, particularly in multivariate analysis. Therefore, future research should expand the sample size, especially for less common subgroups, to enhance the accuracy and reliability of findings. Additionally, the different populations in this study may be influenced by varying treatments or lifestyles, potentially interfering with data accuracy. Future studies should consider a longitudinal research design to track changes more accurately in retinal vascular parameters over time, further validating and deepening the results of this study. The study is also limited to fundus color photography. To develop more effective treatment plans, we plan to expand the scope of data collection to include biochemical data and personal history information. In future, we hope to conduct in-depth analyses of the potential associations between these data and the occurrence of DR, further enriching the research content and enhancing its practical value.

This study provides preliminary evidence revealing the associations between early stages of DR and retinal vascular parameters. However, to comprehensively understand and address the challenges of DR, future research should focus on optimizing and expanding several areas, including disease stage coverage, sample size, population homogeneity, and data collection methods.

## 5 Conclusion

The study findings indicate that elevated levels of FBG, HbA1c, SBP, and DBP facilitate the transition from pre-diabetes to T2DM and simultaneously contribute to the development and progression of mild-to-moderate NPDR. During this process, a decline in the retinal vascular parameter branch_avg_v is observed. Among individuals with T2DM, increased FBG, HbA1c, age, SBP, and DBP exacerbate the occurrence of mild-to-moderate NPDR. In patients with T2DM with mild-to-moderate NPDR, increases in length_avg_v values and decreases in angle_avg_v, asymmetry_avg_v, vessel_length_density, and vessel_density values were noted, with statistically significant differences. These parameter changes serve as warning signs for the occurrence of mild-to-moderate NPDR and can therefore act as potential biomarkers for monitoring vascular changes in pre-diabetic retinopathy among patients with T2DM. The application of these biomarkers in disease diagnosis, follow-up, and treatment processes can lead to more precise disease management. Furthermore, this study explored the specific impact of glucose metabolism status on retinal vascular structure, furnishing a medical rationale for clinicians to implement timely interventions in glycemic disturbances so as to postpone or halt the onset and advancement of DR.

## Data Availability

The raw data supporting the conclusions of this article will be made available by the authors, without undue reservation.

## Abbreviations

Angle_Avg, Average branch angle; Asymmetry_Avg, Average branch asymmetry; Branch_Avg, Average number of branches; CRAE, Central retinal arteriolar equivalent; CRVE, Central retinal venular equivalent; Curvature_Avg, Average tortuosity; D0, Capacity dimension; D1, Entropy dimension; D2, Correlation dimension; DBP, Diastolic blood pressure; DR, Diabetic retinopathy; FBG, Fasting blood glucose; HbA1c, Glycosylated hemoglobin; Length_Avg, Average length; NPDR, Non-proliferative diabetic retinopathy; PDR, Proliferative diabetes retinopathy; SBP, Systolic blood pressure; SL, Singularity length; T2DM, Type 2 diabetes mellitus; Vessel_Density, Vessel area density; Vessel_Length_Density, Overall length density.
